# Asymmetric response of tropical cyclone activity to global warming over the North Atlantic and western North Pacific from CMIP5 model projections

**DOI:** 10.1038/srep41354

**Published:** 2017-01-30

**Authors:** Doo-Sun R. Park, Chang-Hoi Ho, Johnny C. L. Chan, Kyung-Ja Ha, Hyeong-Seog Kim, Jinwon Kim, Joo-Hong Kim

**Affiliations:** 1 School of Earth and Environmental Sciences, Seoul National University, Seoul, Republic of Korea; 2 Guy Carpenter Asia-Pacific Climate Impact Centre, School of Energy and Environment, City University of Hong Kong, Hong Kong, China; 3 Division of Earth Environmental System, Pusan National University, Busan, Republic of Korea; 4 Department of Convergence Study on the Ocean Science and Technology, Korea Maritime and Ocean University, Busan, Republic of Korea; 5 Department of Atmospheric and Oceanic Sciences, University of California, Los Angeles, California, United States; 6 Division of Climate Change, Korea Polar Research Institute, Incheon, Republic of Korea

## Abstract

Recent improvements in the theoretical understanding of the relationship between tropical cyclones (TCs) and their large-scale environments have resulted in significant improvements in the skill for forecasting TC activity at daily and seasonal time-scales. However, future changes in TC activity under a warmer climate remain uncertain, particularly in terms of TC genesis locations and subsequent pathways. Applying a track-pattern-based statistical model to 22 Coupled Model Intercomparison Project Phase 5 (CMIP5) model runs for the historical period and the future period corresponding to the Representative Concentration Pathway 8.5 emissions scenarios, this study shows that in future climate conditions, TC passage frequency will decrease over the North Atlantic, particularly in the Gulf of Mexico, but will increase over the western North Pacific, especially that hits Korea and Japan. Unlike previous studies based on fine-resolution models, an ensemble mean of CMIP5 models projects an increase in TC activity in the western North Pacific, which is owing to enhanced subtropical deep convection and favorable dynamic conditions therein in conjunction with the expansion of the tropics and vice versa for the North Atlantic. Our results suggest that North America will experience less TC landfalls, while northeast Asia will experience more TCs than in the present-day climate.

The impact of future climate change on tropical cyclone (TC) activities has been examined in many previous studies, which closely agrees with one another in terms of the global average. A recent review paper suggested that the global-mean TC genesis frequency is likely to decrease by 6−34% but TC intensities are likely to increase by 2−11% in a warmer climate^1^. However, projected shifts in major genesis locations and TC pathways within each ocean basin vary widely among previous studies^1^,^2^,^3^. Studies based on high-resolution climate models project a large inter-model spread in simulated TC occurrences since model results are sensitive to specifics in model formulations and experimental designs such as physical parameterizations^4^,^5^,^6^,^7^,^8^, resolutions^9^,^10^,^11^,^12^,^13^, and TC detection algorithms^14^,^15^,^16^. Although multi-model ensembles can enhance the statistical confidence in TC projections, it is difficult for individual researchers or institutions to perform a sufficient number of high-resolution climate model runs because of the need for large computing resources. To overcome this limitation, some recent studies suggested alternative approaches based on various statistical techniques, for example, a climate-index-based statistical model^17^,^18^, a trajectory model based on future tropospheric mean flows^19^, genesis potential indices^20^,^21^,^22^,^23^, and random seeds of synthetic storms^24^,^25^. These studies can give us a good inference on future TC changes despite some limitations. One potential weakness is that the empirical relationships derived in these studies for the present climate might differ from those in the future climate. However, considering the advantages of statistical methods such as no requirement for high-computing power and ease of multi-model ensembles based on dozens of climate models, it is worthwhile to apply a statistical method to study future TC activity.

## Results

### Design and performance of statistical model used

**Figure 1:**
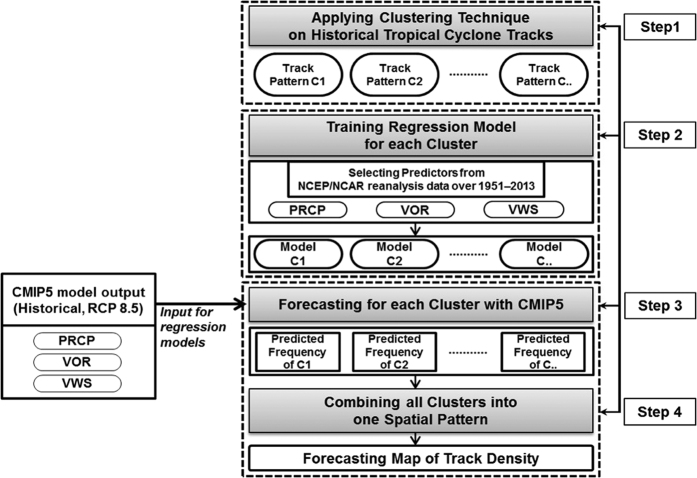
Flowchart of statistical model.

**Figure 2:**
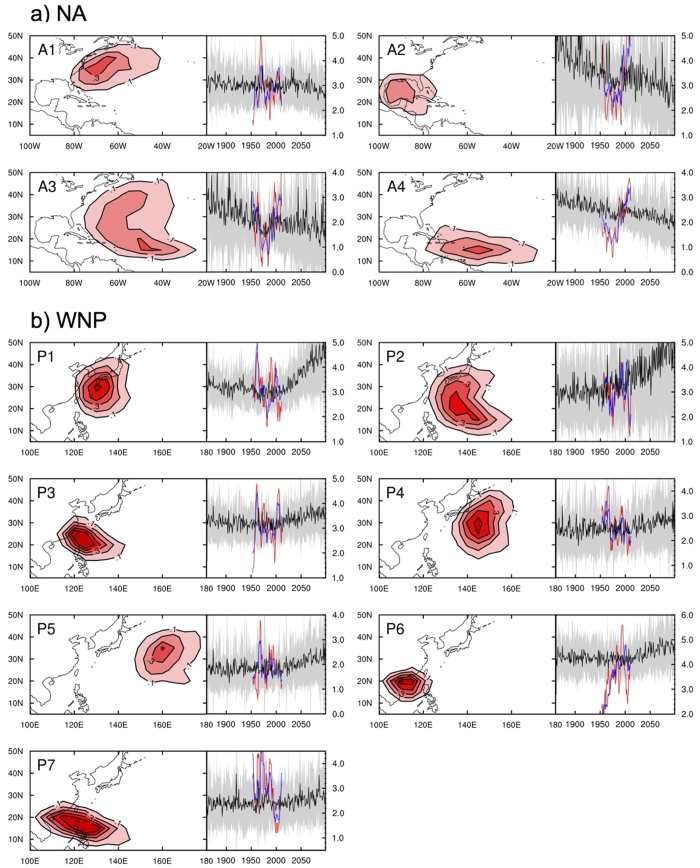
Four and seven clusters of NA and WNP TC tracks (contour interval is 0.1 ratio), their associated time series of observations (red lines), NCEP/NCAR-based retrospective forecast for the period from 1951–2013 (blue lines), and CMIP5-based forecast for the period from 1861–2099 (black lines and gray shading), respectively. For the CMIP5-based forecast, black lines and gray shading indicate the ensemble mean and one-standard-deviation ranges, respectively. The figure was plotted by using NCL version 6.3.0, “The NCAR Command Language (Version 6.3.0) [Software]. (2016). Boulder, Colorado: UCAR/NCAR/CISL/TDD. http://dx.doi.org/10.5065/D6WD3XH5”.

This study employs a track-pattern-based statistical method adopted from the operational statistical-dynamical hybrid system used at the National Typhoon Center of the Korean Meteorological Administration for seasonal forecasting of the North Atlantic (NA) and western North Pacific (WNP) TC activity^26^,^27^. The overall flow of the forecast system was adopted with modifications for the application to long-term climate projections. The model was constructed by the following four steps (Fig. 1). In step 1, historical TC pathways during June–October from 1951–2013 were clustered into four and seven track patterns of NA and WNP TCs, respectively (Fig. 2) by the fuzzy c-means clustering method^28^. The number and general shape of clusters for each basin are almost consistent with previous studies^26^,^27^,^28^,^29^ despite the different analysis periods and clustering methods applied. In other words, the clusters defined here are not so sensitive to change target years and/or clustering methods, and hence the appearance of an entirely new track that is not classified as either one of the present clusters seems unusual. In the future, more TCs may form over the ocean far from the landmass (e.g., the southeast of WNP)^30^,^31^,^32^,^33^. However, we can assume that most of the track patterns making landfall can be categorized into one of the present clusters. TCs formed over far ocean area still have much less chances to hit coastlines even if their landfall changes may increase due to significant track changes. Hence, it is possible to apply the present-day clusters for analyzing future TC track changes as we are more interested in TC impacts that occur on the coastline of countries. In step 2, the interannual variability in the frequency of occurrence of each cluster was used as an explained variable to train a Poisson regression model^26^,^27^ with explanatory variables from the National Centers for Environmental Prediction (NCEP)/National Center for Atmospheric Research (NCEP/NCAR) reanalysis data^34^ for the training period from 1951 to 2013. In step 3, the trained regression models were applied to project the future frequency of each track pattern using the Coupled Model Intercomparison Project Phase 5 (CMIP5) dataset^35^. This study has analyzed projections from 22 CMIP5 models (Supplementary Table S1). In the final step, the projected frequencies of all clusters were combined and transformed into a two-dimensional spatial distribution of TC tracks.

Three variables including convective precipitation, 1000–700 hPa mean relative vorticity, and vertical wind shear were used as predictors to train each cluster’s model on the frequency of occurrence. In the model, convective precipitation represents a thermodynamic condition on TC formation^21^,^22^,^23^, while low-level relative vorticity^36^,^37^,^38^,^39^ and vertical wind shear^39^,^40^,^41^ indicate dynamic factors. The predictor area selected corresponds exactly to the main location of occurrence for each cluster because all of the selected predictors are assumed to directly affect TC formations (Supplementary Figs S1 and S2). It is noted that the significant correlation coefficients between the frequency of occurrence and these three variables occur mostly in the main formation region of each cluster (Supplementary Figs S1 and S2). In the thermodynamic predictors there are several well-known variables such as local sea surface temperatures (SST)^42^, relative SST^17^,^18^,^43^,^44^, potential intensity^20^,^25^,^32^,^45^,^46^,^47^, and convective precipitation^21^,^22^,^23^. As represented by Gray’s genesis parameter^42^, the local SST is not a good predictor for a global warming projection since SST leads to an unrealistic increase in TC genesis^21^,^22^,^23^. For this reason, several studies suggested to use a convective genesis parameter to better display genesis changes in the future^21^,^22^,^23^. In the convective genesis parameter of Gray^21^,^22^,^23^, convective precipitation is utilized as the convective potential, which could replace the thermodynamic potential including ocean thermal energy, moist static stability, and mid-tropospheric humidity. Our statistical model also utilizes convective precipitation instead of SST as one of the predictors to capture thermodynamic changes. In fact, relative SST was also considered as a predictor, but is not included in our model because of overfitting problems which occurred in cases when we added relative SST to our statistical model in conjunction with other dynamic predictors (not shown). This is fairly consistent with the fact that relative SST can alter not only thermodynamic conditions but also dynamic conditions^48^,^49^,^50^,^51^. For example, a higher SST in the North Atlantic than in the Indo-Pacific can reduce the vertical wind shear and result in an increase in hurricane activity^48^.

As shown in the red and blue lines of Fig. 2, our track-pattern-based model is capable of realistically simulating the observed frequency of individual TC clusters at both interannual and decadal timescales when used with large-scale environments from reanalysis data^34^. The correlation coefficients between reanalysis-based retrospective forecasts and observations for each cluster are in the range of 0.49 to 0.70, and all are statistically significant at the 99% confidence level (Supplementary Table S2). Further, a horizontal distribution of the ensemble mean of the reconstructed TC pathways from the 22 CMIP5 model runs reasonably represents the observed patterns from 1986–2005 (Supplementary Fig. S3). These results give confidence that the track-pattern-based model properly represent TC activities under the given large-scale environmental conditions.

### Future projection of TC activity

According to our projections of future TC activities using the track-pattern-based model in conjunction with CMIP5, downward and upward trends in the TC frequency predominates in the NA and the WNP basins from 1861 to 2099, respectively (Fig. 2 and Table 1). All of the NA TC clusters show negative trends although the CMIP5 models yield quite diverse trends, indicating that each cluster is characterized by strong internal variability. The trend of the A1-cluster TCs originating in the subtropical western NA is negligible although it shows a slightly stronger decline after 2050. In fact, less than half of the 22 CMIP5 models show the decline is statistically significant. Otherwise, the frequency of occurrence of the A2, A3, and A4 clusters shows a steady significant decline from 1861 even if their average rate is small (−0.43 per century on average). Among them, the tendency of the A2-cluster is the most notable, and a large number of CMIP5 models support its statistical significance. The weak trends of A2, A3, and A4 are partially attributable to the temporary rebounds in the frequency of occurrence in the late 20^th^ century. These rebounds are imposing because they likely capture the current active period of NA TCs after the mid-1990s^52^,^53^,^54^,^55^. More interestingly, there is no decadal fluctuation in the A1 cluster in both observations and models. The results of the ensemble mean suggest that anthropogenic forcing could have increased formations of NA TCs in the recent decade. Taking an ensemble mean helps to reduce the influence of internal variability in each CMIP5 model results. In turn, anthropogenic forcing will reduce NA TCs as shown in Fig. 2. Unlike in the NA basin, the trends for all WNP TC-clusters are negligible before the 2000s and then become positive and stronger afterward. Their slopes and inter-model spreads vary among clusters. Particularly for P1 and P2, TCs heading to Korea and Japan are the most noticeable with increasing tendencies after the 2000s (i.e., +1.75 per century on average), while for the others the trends are relatively weak with large ensemble spreads. Inter-model differences of P1 are relatively small compared to those of P2s, which suggest that the upward trend in P1 may be confident.

**Figure 3:**
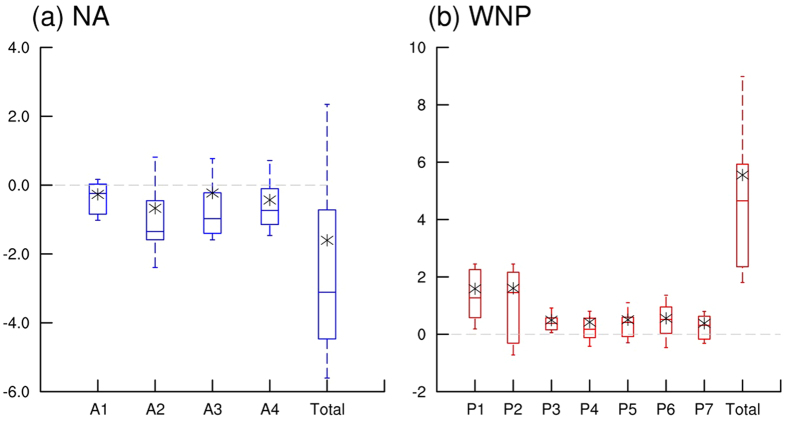
Boxplots of difference in occurrence frequency for each cluster of NA and WNP TCs between the late 21^st^ century (2080–2099) and the present climate period (1986–2005). Uppermost and lowermost black dashed lines indicate upper and lower deciles of differences in TC occurrence frequencies of the models, respectively. Upper, middle, and lower lines of boxes represent upper quartile, median, and lower quartile of the differences, respectively. Asterisks indicate averages of the differences. Gray dashed lines indicate zero values. Red and blue boxes indicate that both of the median and average show same sign of differences, respectively.

To examine future track changes in more detail, all analyses hereafter are based on the differences between the late 21^st^ century (2080–2099) and the present climate period (1986–2005) for individual models as well as for the ensemble mean. Differences between the two periods as shown in the boxplots in Fig. 3 suggest that the majority of CMIP5 models show the same sign in the changes for both basins. In the NA (Fig. 3a), a relatively fast decline of A2 is clearly shown compared to other clusters in all quartiles and averages. This is consistent with previous studies which showed clear decreases in TCs over the Gulf of Mexico^56^,^57^. For each cluster, 14–18 CMIP5 models show negative trends in the TC occurrence frequency, and 10–14 CMIP5 models show declines that are statistically significant at the 95% confidence level (Supplementary Table S3). In terms of the total frequency, i.e., summing up over all four clusters, the number of the NA TCs decreases by 1.6 on average in the late 21^st^ century compared to the present-day climate. Such decreases are found in 17 CMIP5 models with 14 of them statistically significant at the 95% confidence level (Supplementary Table S3). The clear decrease in TC occurrence frequency shown here agrees well with preceding studies on future NA TC activity^58^,^59^,^60^. On the other hand, in the WNP (Fig. 3b), the TCs in the P1 and P2 cluster heading toward Korea and Japan show the largest differences in medians and averages although the lower quartile of P2 is negative due to the large inter-model spread of P2. The substantially increasing P1 and P2 TCs in part corresponds to several studies, which showed that a larger portion of TCs shifts toward the mid-latitude in a warmer climate^19^,^61^. For each cluster, 15–22 CMIP5 models project positive trends, and 6–20 CMIP5 models exhibit a significant increase at the 95% confidence level (Supplementary Table S3). In the total frequency, the number of WNP TCs increases by 5.5 on average. In addition, the upward tendencies are represented by all 22 CMIP5 models with 21 models exhibiting statistical significance at the 95% confidence level (Supplementary Table S3). The increase in the TC occurrence frequency shown here is inconsistent with the results from previous studies^30^,^31^,^32^,^33^ that predicted no change or a decrease in this frequency. However, other studies based on the random synthetic storms^24^,^25^ and genesis potentials^20^,^23^,^25^,^45^ did exhibit future increases, especially for the WNP TCs.

**Figure 4:**
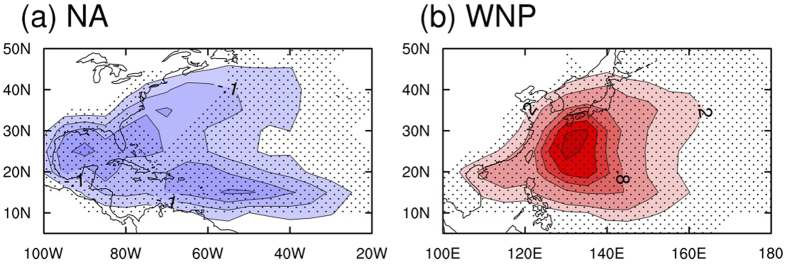
Differences in spatial patterns of TC occurrence frequency over the NA and WNP between the late 21^st^ century (2080–2099) and the present climate period (1986–2005). The contour interval is 0.05 and 0.2 frequency for the NA and WNP, respectively. Dots indicate 15 of 22 CMIP5 models, showing the same signs of averages in differences of TC occurrence frequencies. The size of the grid box is 5° × 5° in a latitude-longitude direction. The figure was plotted by using NCL version 6.3.0, “The NCAR Command Language (Version 6.3.0) [Software]. (2016). Boulder, Colorado: UCAR/NCAR/CISL/TDD. http://dx.doi.org/10.5065/D6WD3XH5”.

Although we can approximate future changes in TC tracks from the time series and boxplots of individual clusters, combining and transforming the time series of each cluster into a spatial map is helpful to see the spatial pattern of the track changes. Figure 4 suggests that the NA TCs appear to approach the North American coastlines less frequently, while the WNP TCs are more likely to strike the coastal regions in East Asia. In the NA, the decrease is most conspicuous in the Gulf of Mexico in which TCs account for most of the TCs striking the United States^29^. In the WNP, the largest increase is observed north of the main TC development regions, indicating a shift in TC activities closer to the northern part of East Asia. The northward shift of TC tracks implies an increase in TC landfalls in the mid-latitude East Asian region. This result is consistent with the recent poleward shift of the TC lifetime maximum intensity during the last three decades in conjunction with the expansion of the tropics^62^,^63^,^64^,^65^. In general, the expansion of tropics is evident under global warming^64^,^65^,^66^ and results in favorable conditions for TC formation in the north of the traditional main development region^62^,^63^. These changes well match our result showing more TCs shifted northward in the WNP.

### Possible mechanisms suggested

**Figure 5:**
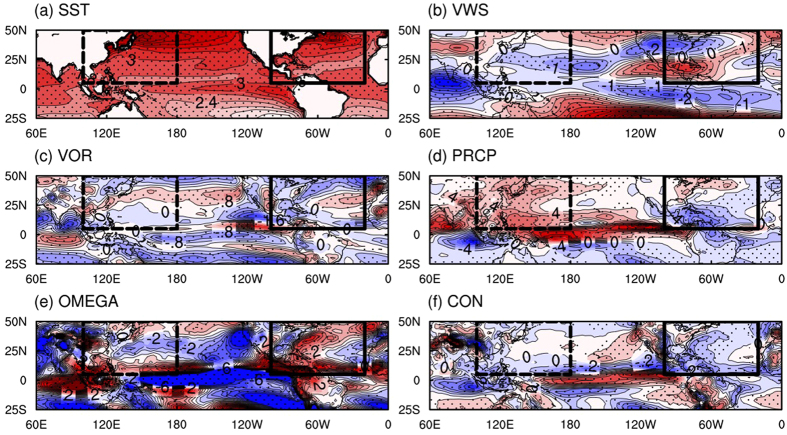
Differences in (**a**) spatial patterns of SST (contour interval is 0.3 °C), (**b**) vertical wind shear between 850- and 200 hPa (VWS, contour interval is 0.5 m s^−1^), (**c**) 1000–700 hPa relative vorticity (VOR, contour interval is 0.4 × 10^−6^ s^−1^), (**d**) convective precipitation (PRCP, contour interval is 2.0 × 10^−1^ mm day^−1^) (**e**) 500-hPa omega (OMEGA, contour interval is 1 × 10^−3^ Pa s^−1^), and (**f**) 1000–700 hPa convergence (CON, contour interval is 0.5 × 10^−6^ s^−1^) between the late 21^st^ century (2080–2099) and the present climate period (1986–2005). Red and blue colors indicate positive and negative values, respectively. Dots indicate 15 of 22 CMIP5 models showing the same signs of averages in differences of TC occurrence frequency. Black boxes with dashed and solid lines indicate WNP and NA ocean basins, respectively. The figure was plotted by using NCL version 6.3.0, “The NCAR Command Language (Version 6.3.0) [Software]. (2016). Boulder, Colorado: UCAR/NCAR/CISL/TDD. http://dx.doi.org/10.5065/D6WD3XH5”.

Changes in the large-scale circulation appear to significantly contribute to the projected changes in TC formations in the NA and WNP basins described above (Fig. 5). According to our statistical model, a considerable portion of changes in TC occurrence is attributable to changes in the convective potential instead of dynamic conditions because the changes in the latter are not as large, particularly in the WNP. Changes in the magnitude of the convective precipitation over the main development regions (MDRs) in the NA (10°N–20°N, 80°W–20°W) and the WNP (10°N–20°N, 100°E–180°) are −0.36 and 0.58 mm per day (i.e., 815 and 1314 kJ m^−2^ in terms of latent heat release), respectively. These changes correspond to a 17% decrease and a 9% increase of convective potential from the present-day climate for each basin. Changes in the other predictors over the NA and WNP MDR regions are: −0.08 × 10^−6^ and −0.09 × 10^−6^ s^−1^ for relative vorticity, and 0.9 and −0.3 m s^−1^ for the vertical wind shear, respectively. In terms of percentages, changes in relative vorticity (−24%) and vertical wind shear (+4%) in the NA are much larger than the changes in relative vorticity (−4%) and vertical wind shear (−3%) in the WNP. In our statistical model, the regression coefficients of convective precipitation for both basins are about two to five times larger than those of relative vorticity and vertical wind shear. This indicates that convective potential is a major driver of TC formation. From another point of view, the strong dependency of the convective potential could be a weakness of our model. However, as shown in Fig. 5, the changes in these three variables consistently support the decrease (increase) in the TC frequency in the NA (WNP). Thus, the overall results are not sensitive to the priority of individual variables.

Despite the high priority of convective potential, the dynamic conditions still have a role to play regarding changes in TC activity. The SST difference pattern suggests that in the later part of this century, the ocean is migrating into a canonical El Niño state although the future SST increase and canonical El Niño patterns are somewhat different from each other in a strict sense^67^. This type of oceanic condition could cause a deep sinking branch (as evidenced by the negative and positive differences of convective precipitation and mid-level omega, respectively) in the Walker-type circulation over the NA, which is set up by the stronger convection in the eastern equatorial Pacific. That is, the enhanced rising motion over the equatorial eastern Pacific could strengthen the downward motion over the NA. Alongside the downward flow associated with decreased convective precipitation, strong divergence and anticyclonic flow appear over the NA. These thermodynamic and dynamic environments could reduce TC occurrence frequency. This reduction of TC frequency can be further enhanced by the positive vertical wind shear difference over the Atlantic, which are driven by El-Niño like warming^45^,^68^,^69^. Note that the enhanced vertical wind shear over the North Atlantic due to global warming was consistently found in the CMIP3 models^45^. All of these signals are predominant in the Gulf of Mexico, and thus they account for the largest reduction of TC occurrence therein. In the WNP, the convective potential significantly increases over the entire basin. The mid-level upward motion also becomes prevalent in the subtropical WNP. These two conditions indicate that the deep convection strengthens the enhanced low-level convergence and cyclonic flow over the subtropics, which could become a large advantage to TC formation there. At the same time, the negative vertical wind shear anomalies, which are the most noticeable near 20°N, could also contribute to an increase in TC frequency. These large-scale changes exactly correspond to the largest increase in TC occurrence frequency in this basin (Fig. 4b). Hence, the thermodynamic and dynamic conditions in the WNP closely match the largest number of TCs over the subtropical basin. Although dynamic conditions are not favorable south of 20°N, thermodynamic conditions (i.e., convective potential) are still favorable. Thus, the tropical cyclone formation can increase south of 20°N even though the increase is not as large as that in the north of 20°N.

## Discussion

This study shows noticeable decreasing and increasing trends in TC occurrence frequencies in the NA and WNP basins, respectively, under warm climate situations projected in CMIP5 experiments. A high degree of consistency among the CMIP5 models yields high confidence in TC activity changes projected in this study. However, in our approach there remains an important question: why do projected changes in the WNP that are based on a statistical model differ from those based on direct TC detections in high-resolution climate model experiments? This is extremely difficult to answer, but it is an important question. Many studies based on fine-resolution models showed decreases in WNP TC frequency and suggested several mechanisms such as increasing static stability, reduced mass flux, and decreasing tropical disturbance^30^,^31^,^32^,^33^. Even if all of these mechanisms strongly support their results, they also must be explained by large-scale circulation changes. Many previous studies showed a significant future increase in TC genesis potential over the WNP using genesis indices^20^,^23^,^25^,^45^. Similarly, our analyses show increasing convective potential and mid-level upward motion. Dynamic conditions also become favorable for TC formation over the subtropical WNP in the ensemble mean of CMIP5 although they appear to have played a minor role in altering TC occurrence frequency in our statistical model. The consistency between our results and previous studies gives us more confidence in our results. Hence, this study can provide an additional reference of future TC activity despite differences from previous studies^30^,^31^,^32^,^33^.

## Data and Methods

### Data

The historical best-track TC data used for the NA and WNP are issued by the National Hurricane Center^70^ and the Regional Specialized Meteorological Center in Tokyo, respectively. The data include six-hourly information on individual TCs such as their maximum wind speeds, minimum pressures, and geographical locations. The tropospheric variables used as explanatory variables include convective precipitation, 1000–700 hPa mean relative vorticity, and vertical wind shear between 850 and 200 hPa. The observational data are derived from the NCEP/NCAR reanalysis on a 2.5° × 2.5° longitude–latitude grid^34^, while the projection data are from 22 CMIP5 models based on historical and representative concentration pathway 8.5 scenarios^35^ (listed in Supplementary Table S1) with diverse horizontal resolutions among the models. The SST data are from the Hadley Centre SST data set version 1, on a 1° × 1° longitude–latitude grid^71^. The Hadley SST and CMIP5 data are horizontally re-gridded to 2.5° × 2.5° prior to analysis.

### Predictor calculation

Here, we used anomalies based on the present climate. This method made it possible to mitigate biases between model and real climates. The time series of each predictor were calculated by the following equation:


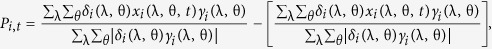


where 
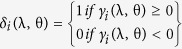
 for convective precipitation and relative vorticity, 
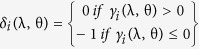
 for vertical wind shear, [] indicates the time mean for the period from 1986–2005, *x*_*i*_(λ, θ, t) indicates the value of variable *i*, of predictors at a given year (t), latitude (θ), and longitude (λ) within the predictor-area, and *γ*_*i*_(λ, θ) is the correlation coefficient of the variable *i* with the occurrence frequency of the cluster of interest. The characteristics of delta *δ*_*i*_(λ, θ) are determined by following a favorable condition of the variable *i* for TC development. For example, the delta of the vertical wind shear is −1 when *γ*_*i*_(λ, θ) ≤ 0, which implies that the reduced vertical wind shear favors TC occurrence of the cluster. A similar approach is applied to the other variables. Finally, after dividing the standard deviation for the present climate period (i.e., 1986–2005), *P*_*i,t*_ is used as a predictor for variable *i*.

## Additional Information

**How to cite this article:** Park, D.-S. R. *et al*. Asymmetric response of tropical cyclone activity to global warming over the North Atlantic and western North Pacific from CMIP5 model projections. *Sci. Rep.* **7**, 41354; doi: 10.1038/srep41354 (2017).

**Publisher's note:** Springer Nature remains neutral with regard to jurisdictional claims in published maps and institutional affiliations.

## Supplementary Material

Supplementary Tables and Figures

## Figures and Tables

**Table 1:** Trends of ensemble mean for the periods 1861–2099 and 1986–2099 (occurrence frequency per century).

	1861–2099	1986–2099
A1	−0.1 (9, 8)	−0.3 (9, 9)
A2	−0.7 (17, 17)	−0.6 (15, 15)
A3	−0.4 (15, 15)	−0.1 (14, 13)
A4	−0.4 (15, 14)	−0.4 (14, 14)
P1	0.5 (18, 18)	1.8 (20, 19)
P2	0.8 (14, 14)	1.7 (12, 11)
P3	0.2 (12, 10)	0.5 (12, 9)
P4	0.2 (10, 7)	0.5 (8, 8)
P5	0.2 (13, 13)	0.6 (11, 10)
P6	0.2 (10, 10)	0.6 (13, 12)
P7	0.1 (9, 8)	0.4 (9, 9)

The two numbers in parentheses indicate the number of 22 CMIP5 models whose trends have the same signs with the ensemble means and statistical significance at the 90% (left) and 95% (right) confidence levels, respectively, evaluated with the Student’s *t*-test.
