# Supplementary material for: Asymmetric response of tropical cyclone activity to global warming over the North Atlantic and western North Pacific from CMIP5 model projections
Source: Sci Rep. 2017 Jan 30;7:41354. doi: 10.1038/srep41354 (PMC5278409; doi:10.1038/srep41354)
Supplement: Supplementary Tables and Figures [file srep41354-s1.pdf]

# Asymmetric response of tropical cyclone activity to global warming over the North Atlantic and the western North Pacific from CMIP5 model projections

Doo-Sun R. Park[1], Chang-Hoi Ho[1,*], Johnny C. L. Chan[2], Kyung-Ja Ha[3], Hyeong-Seog Kim[4], Jinwon Kim[5], and Joo-Hong Kim[6]

[1]School of Earth and Environmental Sciences, Seoul National University, Seoul, Republic of Korea

[2]Guy Carpenter Asia-Pacific Climate Impact Centre, School of Energy and Environment, City University of Hong Kong, Hong Kong, China

[3]Division of Earth Environmental System, Pusan National University, Busan, Republic of Korea

[4]Ocean Science and Technology School, Korea Maritime and Ocean University, Busan, Republic of Korea

[5]Department of Atmospheric and Oceanic Sciences, University of California, Los Angeles, California, United States

[6]Division of Climate Change, Korea Polar Research Institute, Incheon, Republic of Korea

[*]Corresponding author: Chang-Hoi Ho, hoch@cpl.snu.ac.kr

November 2016

Scientific Reports (revised)

**Supplementary Table S1.** 22 CMIP5 models used in this study.

| Models used | |
|---|---|
| CESM1-CAM5 | IPSL-CM5B-LR |
| CMCC-CMS | IPSL-CM5B-MR |
| CNRM-CM5 | MIROC5 |
| CanESM2 | MIROC-ESM |
| GFDL-ESM2G | MIRCO-ESM-CHEM |
| GFDL-ESM2M | MPI-ESM-LR |
| GISS-E2-H | MPI-ESM-MR |
| GISS-E2-R | MRI-CGCM3 |
| HadGEM2-AO | NorESM1-ME |
| IPSL-CM5A-LR | NorESM1-M |
| bcc-csm1-1 | Inmcm4 |

**Supplementary Table S2.** Correlation coefficient between interannual time series of observation and NCEP/NCAR-based retrospective forecast in TC occurrence frequency for each cluster based on the leave-one-out cross-validation. All of correlation coefficients are statistically significant at the 99% confidence level.

| Basin | Cluster type | R |
|---|---|---|
| NA | A1 | 0.51 |
| | A2 | 0.64 |
| | A3 | 0.54 |
| | A4 | 0.50 |
| WNP | P1 | 0.51 |
| | P2 | 0.70 |
| | P3 | 0.61 |
| | P4 | 0.60 |
| | P5 | 0.55 |
| | P6 | 0.49 |
| | P7 | 0.62 |

**Supplementary Table S3.** The number of 20 CMIP5 models representing the same signs in the differences of basin-total and each-cluster TC occurrence frequency between the late 21st century (2080–2099) and the present climate period (1986–2005). Two numbers in parentheses indicate the number of 22 CMIP5 models whose differences are statistically significant at the 90% (left) and 95% (right) confidence levels, respectively, evaluated with a Monte-Carlo method.

| | Positive sign | Negative sign |
|---|---|---|
| A1 | 8 (3,2) | 14 (11,10) |
| A2 | 4 (4,3) | 18 (15,14) |
| A3 | 5 (4,4) | 17 (14,13) |
| A4 | 6 (4,4) | 16 (15,14) |
| $A_{basin\text{-}total}$ | 5 (4,4) | 17 (14,14) |
| P1 | 22 (20,20) | 0 (0,0) |
| P2 | 16 (13,12) | 6 (2,2) |
| P3 | 21 (15,10) | 1 (1,0) |
| P4 | 15 (8,7) | 7 (3,0) |
| P5 | 15 (11,11) | 7 (1,1) |
| P6 | 18 (13,13) | 4 (2,2) |
| P7 | 15 (12,6) | 7 (1,1) |
| $P_{basin\text{-}total}$ | 22 (21,21) | 0 (0,0) |

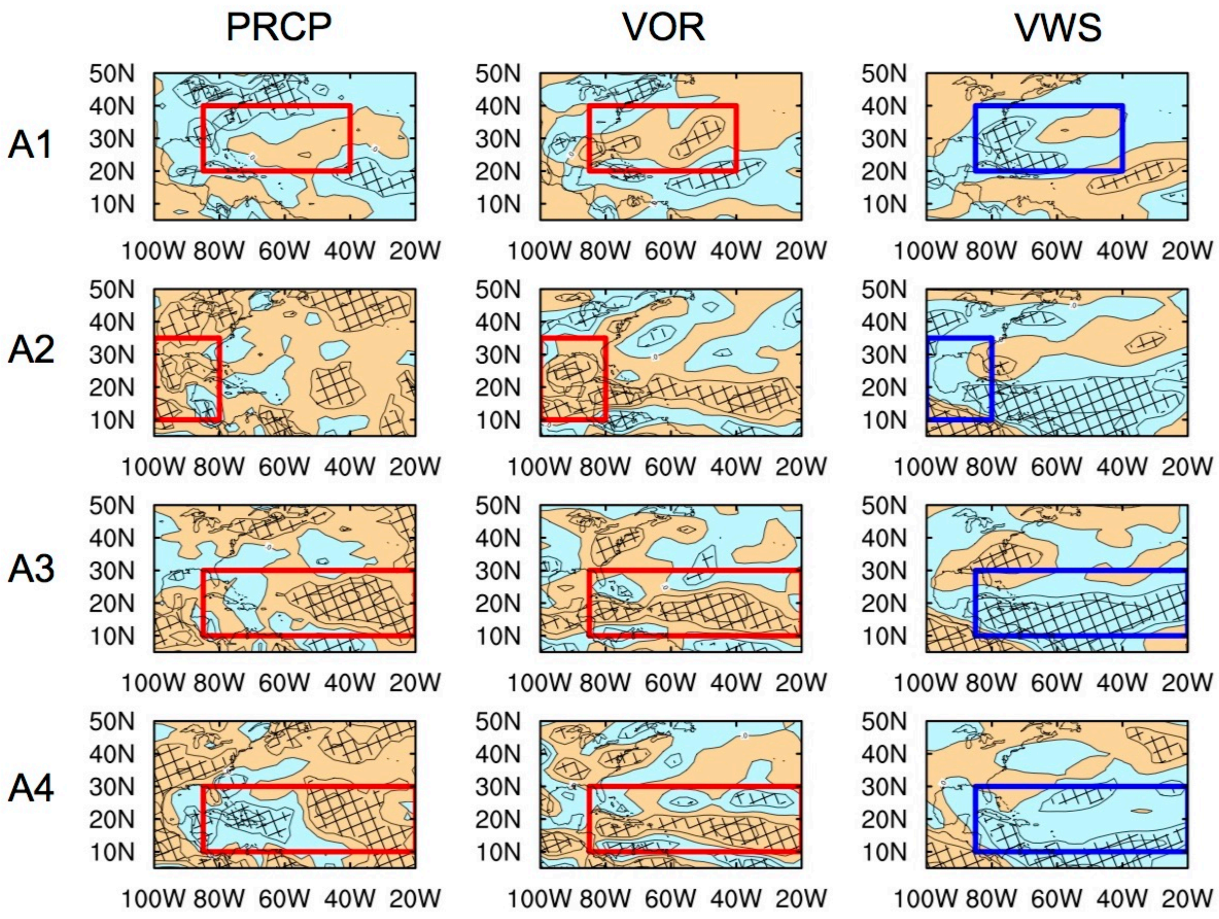


**Supplementary Figure S1.** Correlation–coefficient maps of convective precipitation (PRCP, leftmost column), 1000–700 hPa relative vorticity (VOR, center column), and vertical wind shear between 850- and 200 hPa (VWS, rightmost column) with each NA TC cluster's interannual time series in occurrence frequency. Red (blue) boxes indicate that positive (negative) correlations are only considered when calculating the predictor time series. Hatches indicate correlation coefficients are statistically significant at the 90% confidence level. The figure was plotted by using NCL version 6.3.0, "The NCAR Command Language (Version 6.3.0) [Software]. (2016). Boulder, Colorado: UCAR/NCAR/CISL/TDD. http://dx.doi.org/10.5065/D6WD3XH5".

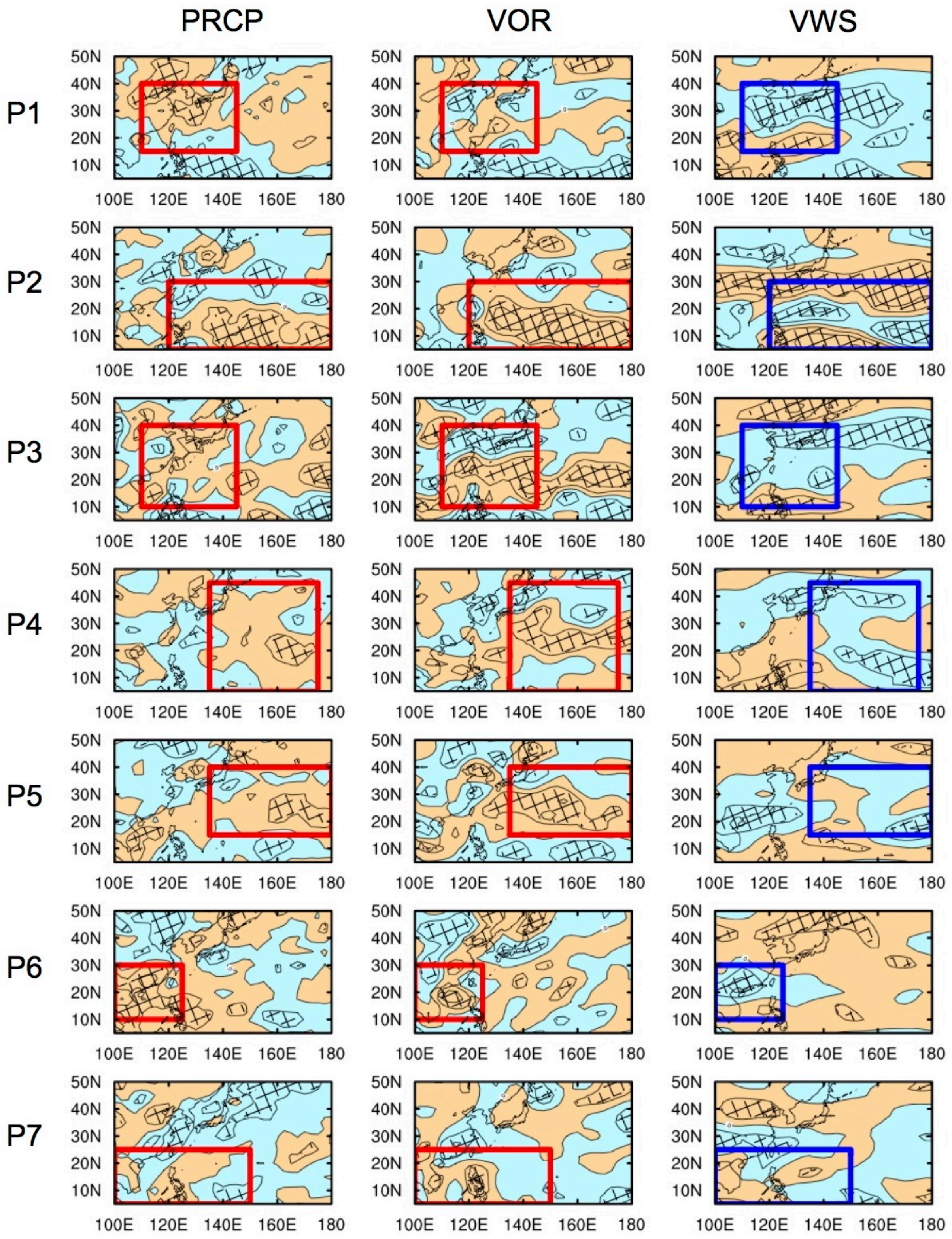


**Supplementary Figure S2.** Same as S. Fig. S1, but for WNP TC clusters.

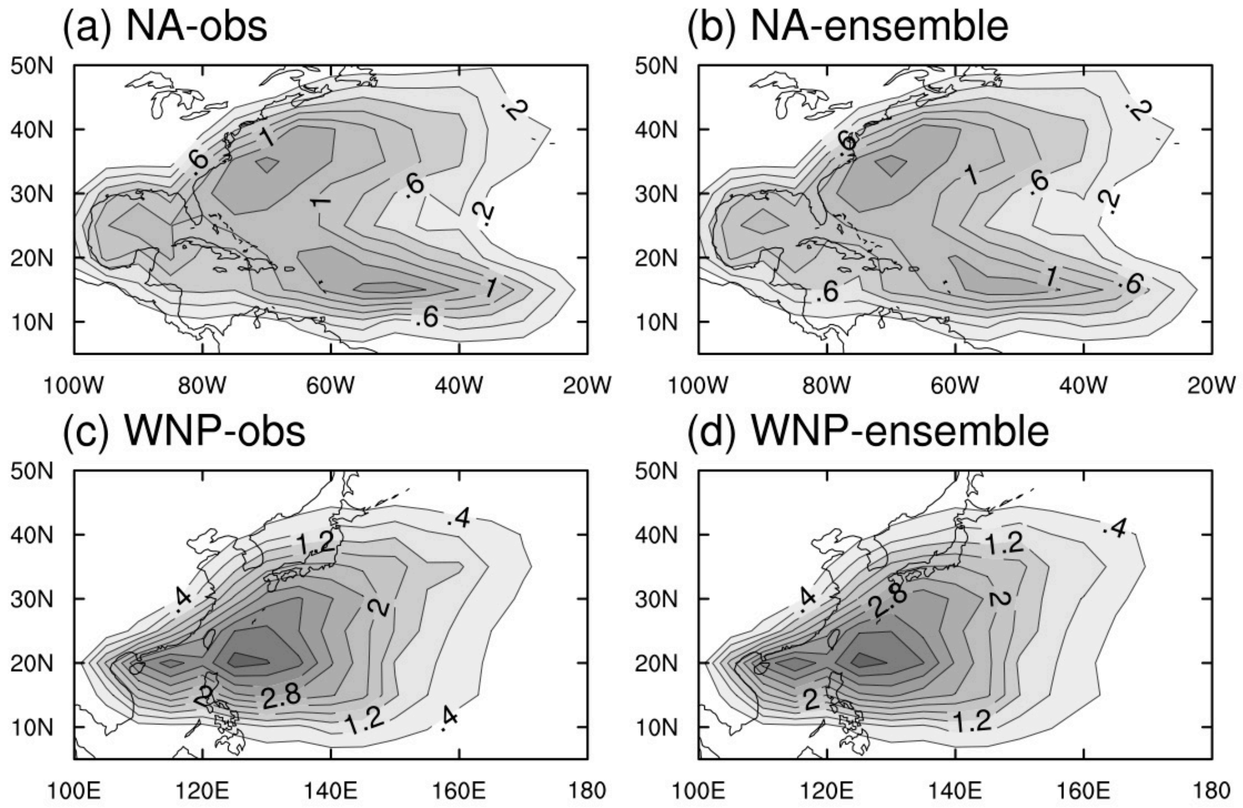


**Supplementary Figure S3.** Climatological spatial patterns of NA and WNP TC occurrences in observations and CMIP5-ensemble mean for the present climate period (1986–2005). The size of the grid box is 5°×5° in a latitude-longitude direction. The figure was plotted by using NCL version 6.3.0, "The NCAR Command Language (Version 6.3.0) [Software]. (2016). Boulder, Colorado: UCAR/NCAR/CISL/TDD. http://dx.doi.org/10.5065/D6WD3XH5".
